# *Pharmacokinetic and Pharmacodynamic Integration* and Resistance Analysis of Tilmicosin Against *Mycoplasma gallisepticum* in an *In Vitro* Dynamic Model

**DOI:** 10.3389/fphar.2019.00670

**Published:** 2019-06-12

**Authors:** Zilong Huang, Yuzhi Wu, Zichong Zhou, Xirui Xia, Xiaoyan Gu, Qinren Cai, Xiangguang Shen, Hong Yang, Huanzhong Ding

**Affiliations:** ^1^Guangdong Key Laboratory for Veterinary Drug Development and Safety Evaluation, South China Agricultural University, Guangzhou, China; ^2^Technical Center for Inspection and Quarantine, Zhuhai Entry–Exit Inspection and Quarantine Bureau, Zhuhai, China; ^3^School of Life Science and Engineering, Foshan University, Foshan, China

**Keywords:** tilmicosin, *Mycoplasma gallisepticum*, dynamic model, pharmacokinetics, pharmacodynamics, resistance, chronic respiratory disease

## Abstract

*Mycoplasma gallisepticum* is the major pathogen causing chronic respiratory disease in chickens. In the present study, we successfully established a one-compartment open model with first-order absorption to determine the relationship between tilmicosin pharmacokinetic and pharmacodynamic (PK/PD) indices and *M. gallisepticum* in *in vitro*. The aim was to simulate the PK/PD of tilmicosin against *M. gallisepticum* in lung tissues. The results of static time-killing curves at constant drug concentrations [0–64 minimum inhibitory concentration (MIC)] showed that the amount of *M. gallisepticum* was reduced to the limit of detection after 36 h when the drug concentration exceeded 1 MIC, with a maximum kill rate of 0.53 h^-1^. In dynamic time-killing studies, tilmicosin produced a maximum antimycoplasmal effect of 6.38 Log_10_ CFU/ml reduction over 120 h. The area under the concentration–time curve over 24 h divided by the MIC (AUC_24h_/MIC) was the best PK/PD parameter to predict the antimicrobial activity of tilmicosin against *M. gallisepticum* [R^2^ = 0.87, compared with 0.49 for the cumulative time that the concentration exceeds the MIC (%T > MIC)]. Therefore, tilmicosin showed concentration-dependent activity. Seven *M. gallisepticum* strains (M1–M7) with decreased susceptibility to tilmicosin were isolated from seven dose groups. These strains of *M. gallisepticum* had acquired resistance to erythromycin as well as to tylosin. However, no change in susceptibility to amikacin and doxycycline was observed in these strains. Gene mutation analysis was performed on the basis of annotated single nucleotide polymorphisms using the genome of strain S6 as the reference. For strain M5, a G495T mutation occurred in domain II of the 23S *rrnA* gene. In strain M3, resistance was associated with a T854A mutation in domain II of the 23S *rrnB* gene and a G2799A mutation in domain V of 23S *rrnB*. To the best of our knowledge, these tilmicosin resistance-associated mutations in *M. gallisepticum* have not been reported. In conclusion, tilmicosin shows excellent effectiveness and concentration-dependent characteristics against *M. gallisepticum* strain S6 *in vitro*. Additionally, these results will be used to provide a reference to design the optimal dosage regimen for tilmicosin in *M. gallisepticum* infection and to minimize the emergence of resistant bacteria.

## Introduction

Mycoplasmosis is commonly caused by the pathogen *Mycoplasma gallisepticum*, which has characteristics of a small volume, no cell wall, and difficult *in vitro* cultivation. Pathogenic mycoplasmas often cause chronic respiratory disease and infectious sinusitis disease, with clinical signs including cough, depression, sinusitis, nasal discharge, and keratoconjunctivitis, by colonizing the mucosal surface of the respiratory tract (Levisohn and Kleven, 2000). Once flocks of chickens are infected with mycoplasma, *M. gallisepticum* is difficult to eradicate because of its vertical transmission ability. *M. gallisepticum* infection has caused large economic loses in the chicken breeding industry worldwide. *M. gallisepticum* infections are often accompanied by various other pathogen infections (Gunther et al., 2008), especially in areas with poor sanitation and high-density breeding, which results in aggravation of the disease. Antimicrobial chemotherapy is the preferred method to control the development of mycoplasma infection (Prescott and Baggot, 1988). Tilmicosin, a macrolide antibiotic for veterinary use, has a long elimination half-life and accumulates at high concentrations in lung tissue (Jianzhong et al., 2005; Abu-Basha et al., 2007; Zhang et al., 2016). In addition, tilmicosin possesses a fairly broad efficacy spectrum, especially toward mycoplasma (Charleston et al., 1998; Ziv et al., 2010). The unique nature of tilmicosin is ideal for the treatment of *M. gallisepticum* infections.

In recent years, *in vitro* pharmacokinetic and pharmacodynamic (PK/PD) models have been widely used as important scientific research methods to optimize dose regimens, classify the antibacterial activity of drugs, and prevent the occurrence of resistant microbes (Vinks et al., 2014). Importantly, the magnitudes of the PK/PD indices for efficacy in *in vitro* model have been shown to be very similar in animal infection models (Andes and Craig, 2002; Bonapace et al., 2002; Booker et al., 2005). An *in vitro* PK/PD model not only can accurately emulate the process of drug concentration change (fitted to a compartment model) in animals, but also can eliminate the differences among animal species (Wang et al., 2013). It is difficult to establish an *in vivo* infection model of *M. gallisepticum*. Therefore, establishing an *in vitro* dynamic infection model seems to be a feasible method to evaluate the effect of tilmicosin against *M. gallisepticum*.

The mechanism of drug resistance to macrolide antibiotics mainly includes changing the target molecule of drug binding, inactivation of enzyme activity, and active efflux mechanisms (Weisblum, 1995). Most previous studies reported that resistance to macrolides was associated with mutations within in the *rplD* and *rplV*, genes encoding ribosomal proteins L4 and L22, or domain II or V of the 23S rRNA genes (Vester and Douthwaite, 2001; Gerchman, 2011).

The aims of this study were to determine the antibacterial activity of tilmicosin against *M. gallisepticum* by establishing an *in vitro* PK/PD model that simulated the pharmacokinetics of tilmicosin in lung tissue, and thus to investigate the mechanism of resistance. This model can provide a reference for optimizing the antibacterial dosing regimen.

## Materials and Methods

### Materials

The *M. gallisepticum* standard strain S6 was obtained from the Chinese Veterinary Microorganism Culture Collection Center (Beijing, China). Tilmicosin (80.04%), tylosin (82.6%), erythromycin (85.0%), tiamulin (99.0%), doxycycline (85.8%), and enrofloxacin (99.0%) were kindly supplied by Guangdong Dahuanong Animal Health Products (Xincheng, China); and amikacin (99.0%) and lincomycin (84.6%) were purchased from Guangdong Puboxing Animal Health Products, and stored at −80°C before using. *M. gallisepticum* artificial medium base was purchased from Qingdao Hope Biological Technology (Qingdao, China). Nicotinamide adenine dinucleotide (NADH) and cysteine were purchased from Guangzhou Prob Information Technology.

### Inoculum Preparation

The culture system comprised 30 ml of a logarithmic growth culture of *M. gallisepticum* and 300 ml of broth incubated in a carbon dioxide incubator for 36 h. Six tubes of 50 ml of logarithmic growth culture were concentrated using centrifugation (4,000 × *g* for 20 min). After centrifugation, the supernatant was discarded. The cell pellet was suspended in 1 ml of broth and placed in a 10-ml centrifuge tube. The cells were concentrated by centrifugation (4,000 × *g* for 20 min) again. The cell pellet was resuspended in 10 ml of fresh broth. The final concentration of the culture was about 3 × 10^9^ colony forming units (CFU)/ml.

### Determination of the Minimum Inhibitory Concentration

The MIC of tilmicosin against *M. gallisepticum* strain S6 was determined using a modified MIC assay method reported by Tanner and Wu (1992). Briefly, cultures at the logarithmic growth phase were diluted with medium to 10^5^ and 10^7^ CFU/ml. Twofold serial dilutions were then performed. The concentration of tilmicosin in a 96-well plate was 0.000625–0.16 μg/ml. *M. gallisepticum* at 10^5^ or 10^7^ CFU/ml was added into each well in an equal volume of medium. At the same time, a growth control (inoculum in absence of antimicrobials), an end-point control (blank medium at pH 6.8), and a sterility control (sterile medium at pH 7.8) were also used. Plates were cultured in 37°C, 5% CO_2_ in a humidified carbon dioxide incubator until the growth group and the end-point control were of the same color. The minimal drug concentration that caused no color change was defined as the MIC.

According to the method described by Hannan et al. (1989), 100 μl of cells at 10^9^ CFU/ml was spread on plates containing a series of tilmicosin concentrations ranging from 0.01 to 0.64 μg/ml. Samples (10 μl) of cultures with an inoculum of 10^5^ and 10^7^ CFU were also applied to the drug plates. A growth group control was also used comprising cells spread on plates lacking the drug. The plates were incubated for 7 days. The lowest concentration without *M. gallisepticum* growth was determined as the MIC. All experiments were performed in triplicate.

### Time-Kill Curve Studies

The concentration of tilmicosin was in a certain range (1/2, 1, 2, 4, 8, 16, 32, and 64 the MIC), and the MIC value determined for an *M. gallisepticum* inoculum of 10^7^ CFU/ml was applied. In a penicillin bottle, the culture system comprised 3.86 ml of blank medium, 0.04 ml of 10 times the final drug concentration, and 0.1 ml of logarithmic *M. gallisepticum*. The final inoculation was 10^7^ CFU/ml. The penicillin bottles were cultured for 48 h at 37°C, with 5% CO_2_. Aliquots (100 μl) of the culture were taken from each bottle at 0, 1, 3, 6, 9, 12, 24, 36, and 48 h to detect the population of *M. gallisepticum*. A growth control (not exposed to the drug) and a sterility control (medium at pH 7.8 without the drug and *M. gallisepticum*) were included. Samples (10 μl) of the culture were diluted with medium using a 10-fold serial dilution method and plated onto blank agar plates at each time point. Plates were incubated for at least 7 days in a 37°C, 5% CO_2_ humidifier incubator. All experiments were performed in triplicate.

### Description of the Model

**Figure 1:**
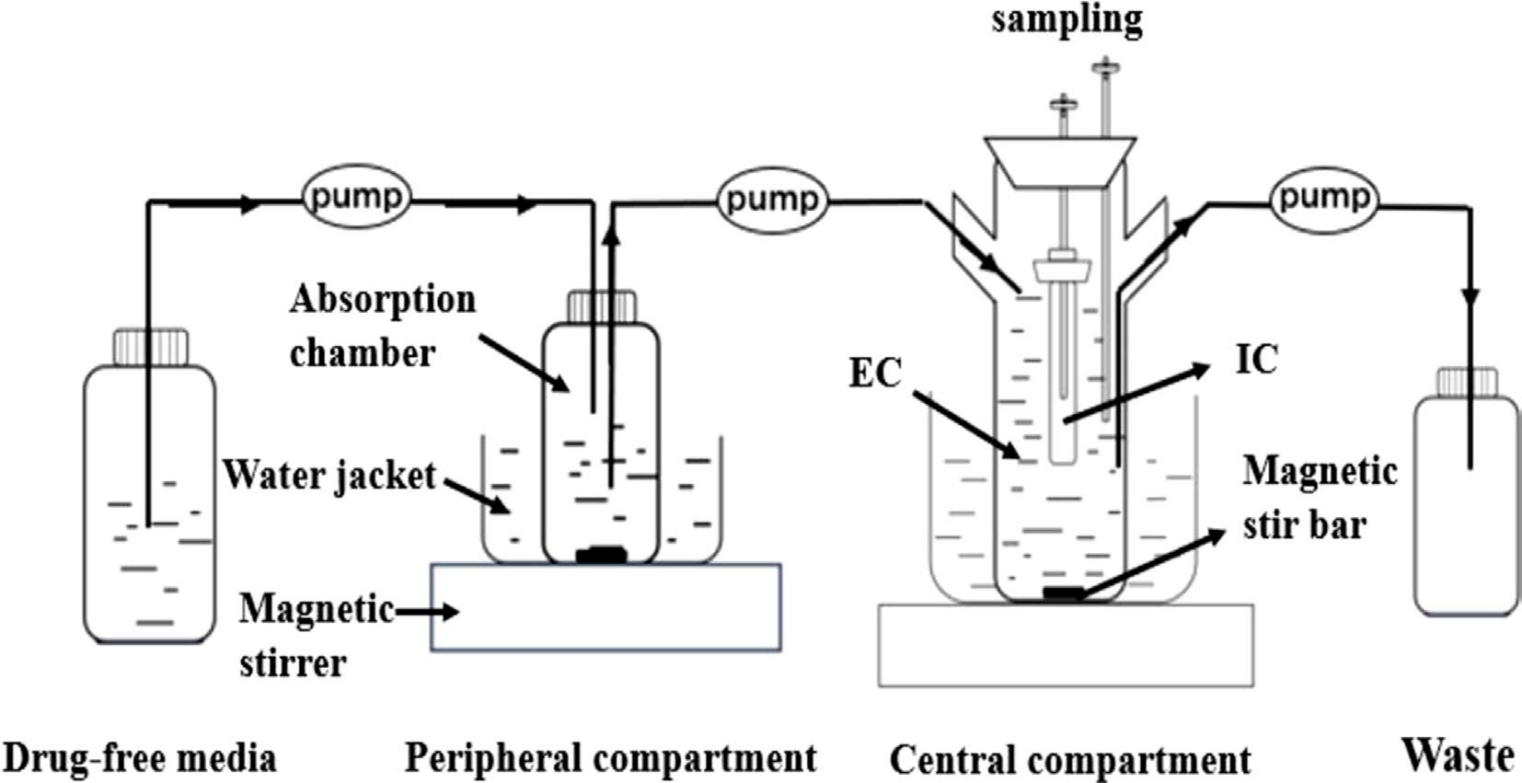
The *in vitro* model that simulates the pharmacokinetics of tilmicosin in lung tissues of the *Mycoplasma gallisepticum*-infected chickens and determines a drug’s effect on the growth and susceptibility of *M. gallisepticum*. EC, external compartment; IC, internal compartment.

In the present study, we developed a modified version of a previously described *in vitro* PD model (Li et al., 2016), comprising a one-compartment open model with first-order absorption of pharmacokinetics for tilmicosin. The model was applied according the outline in shown **Figure 1**. Briefly, the model system consisted of three compartments, a multi-channel peristaltic pump, and constant temperature magnetic stirrers. One of compartments was the absorption chamber containing 61.54 ml of drug-containing medium, which acted as administration site. The second compartment was the central chamber, which comprised 500 ml of sterile medium [external compartment (EC)] and a 10-ml volume dialysis tube [internal compartment (IC)]. The two chambers above were continuously diluted using a peristaltic pump. The third compartment was the reserve chamber, which was used to provide fresh medium. At the same time, an empty bottle was used to collect waste. The role of the constant temperature magnetic stirrer was to thoroughly mix the drug with medium using a rotor at the bottom of each compartment and to maintain the optimal growth temperature (37°C) for *M. gallisepticum*. Operation of peristaltic pumps completed the process of continuously changing the drug at a constant rate. This experiment was based on accurately simulating the drug concentration in lung tissue. According to the parameters determined in our laboratory (unpublished), the flow rate of the peristaltic pumps was set at 0.13 ml/min.

### *In Vitro* Pharmacokinetic and Pharmacodynamic Model and Dosing Regimens

Selection of the model parameters depended on the colonization site of *M. gallisepticum* and the PK characteristics of tilmicosin in chickens. The parameter values of absorption half-life, elimination half-life, and apparent volume of distribution in the lung were 5.37 h, 42.83 h, and 5.6 L/kg, respectively. According to the clinically recommended dose, seven different dose groups (1, 2.5, 5, 7.5, 10, 15, and 20 mg) were designed for the *in vitro* dynamic model. To ensure the stability of *M. gallisepticum*, 0 h of the model was defined as the state of the IC at 12 h after inoculation 2 × 10^9^ CFU/ml of *M. gallisepticum* in 10 ml of medium. At time zero, the drug was injected into the absorption chamber, accelerating the speed of the magnetic stirrers to achieve a rapid balance between inside and outside of the dialysis membrane simultaneously.

Samples (1.5 ml) were collected from the EC at 1, 3, 6, 9, 12, 24, 36, 48, 72, 96, 120, 144, and 168 h after administration, and then stored at −20°C until analysis. Samples (100 μl) were taken from the IC before dosing and at 6, 12, 24, 36, 48, 72, 96, 120, 144, and 168 h after administration. The amount of *M. gallisepticum* and its susceptibility were detected using the collected samples.

### Quantification of Tilmicosin in the Medium

The concentrations of tilmicosin in the medium were analyzed using high-performance liquid chromatography–tandem mass spectrometry (HPLC-MS/MS). Referring to the standard of Announcement No. 1025 of the Ministry of Agriculture in China, pretreatment of the samples was optimized and acetonitrile was selected as the only extractant. The mobile phase consisted of solution A (water with 0.1% formic acid, V/V) and solution B (acetonitrile) at 0.25ml/min flow rate. The gradient elution was: 0–1.5 min, 10% B; 1.5–6 min, 95% B; 6–6.5 min, 5% B; and 6.5–12.5 min, 5% B. The injection volume was 5 μl. The PK parameters were calculated using WinNonlin Software (version 6.1; Pharsight Corporation, Mountain View, Sunnyvale, CA, USA).

### Time–Killing Curve Fitting and Analysis

The bactericidal rate can be used to evaluate the antibacterial effects of antimicrobial agents on microorganisms. The greater the kill rate, the stronger the bactericidal effect of the drug on the bacteria (Nielsen et al., 2007; Ferro et al., 2015). Evaluation of the kill rate was performed at different time intervals (0–6, 0–9, 1–6, 1–9, and 3–9 h), in which linear regression was used to determine the slope for each concentration. Linear regression can take into consideration the overall situation of the time different points in the whole time interval. The relationship between the average kill rate of the five time intervals and each concentration was analyzed using the sigmoid maximum effect (*E*_max_) model employing WinNonlin (version 6.1, Pharsight Corporation). The *E*_max_ model could be described using the following equation:

$$E=E_{0}+\frac{(E_{\max}-E_{0})\times C_{e}^{N}}{EC_{50}^{N}+C_{e}^{N}}$$

where *E* is the kill rate, *E*_0_ and *E*_max_ are the baseline value and maximum value of the efficacy, respectively, *C* *_e_* is the tilmicosin concentration, *EC* *_50_* is the concentration of tilmicosin when 50% of the maximum change (*E*_max_–*E*_0_) was reached, and *N* is the Hill coefficient, which was used to reflect the curve’s slope.

### Susceptibility Testing of *M. gallisepticum*

The samples in the IC at the last time point were cultured to the logarithmic growth phase, and then 10 μl of appropriate dilution with 10^7^ CFU/ml was dropped on the surface of drug plates containing 1 × MIC concentration. After 7 days of culture, the recovered colonies on the plate were transferred to blank medium and continuously passaged five times until they grew stably. The MICs of the strains were determined by the microdilution method, as described in the section Determination of the Minimum Inhibitory Concentration. Compared with strain S6, the MIC values remained high for selected strains with reduced sensitivity. The selected strains were also tested for sensitivity to other antimicrobial agents, including tylosin, erythromycin, tiamulin, doxycycline, enrofloxacin, amikacin, and lincomycin.

### Pharmacokinetic–Pharmacodynamic Integration and Modeling

By integrating the PK parameters and the MIC value *in vitro*, two important PK/PD indices (%T > MIC and AUC_24h_/MIC) were calculated. Using the WinNonlin software to fit the correlation between antimicrobial activity against *M. gallisepticum* and PK/PD indices, we fitted data using the inhibitory sigmoid *E*_max_ model, as described below.

$$E=E_{\max}-\frac{(E_{\max}-E_{0})\times C_{e}^{N}}{EC_{50}^{N}+C_{e}^{N}\text{​}}$$

where *E* is the antimycoplasmal effect; *E*_max_ is the change in the amount of *M. gallisepticum* in the control group at a 24-h interval; *E*_0_ is the largest antimycoplasmal effect, determined as log_10_CFU/ml reduction at the same interval; *C* *_e_* represents the PK/PD indices (%T > MIC and AUC_24h_/MIC); *EC* *_50_* is the corresponding PK/PD index value when the antimycoplasmal effect reaches e 50% of the maximum antibacterial effect; *N* is the Hill coefficient that describes the steepness of the PK/PD indices-effect curve; and *R* *^2^* was calculated for each assay.

## Results

### Minimum Inhibitory Concentration

The MIC values of tilmicosin with inoculums of 10^5^ and 10^7^ CFU/ml were 0.01 and 0.02 μg/ml, respectively, using the microdilution method. For inoculums of 10^5^, 10^7^, and 10^9^ CFU, the MIC values were 0.02, 0.04, and 0.16 μg/ml using the agar dilution method, respectively.

### Time-Killing Curve Fitting and Analysis

**Figure 2:**
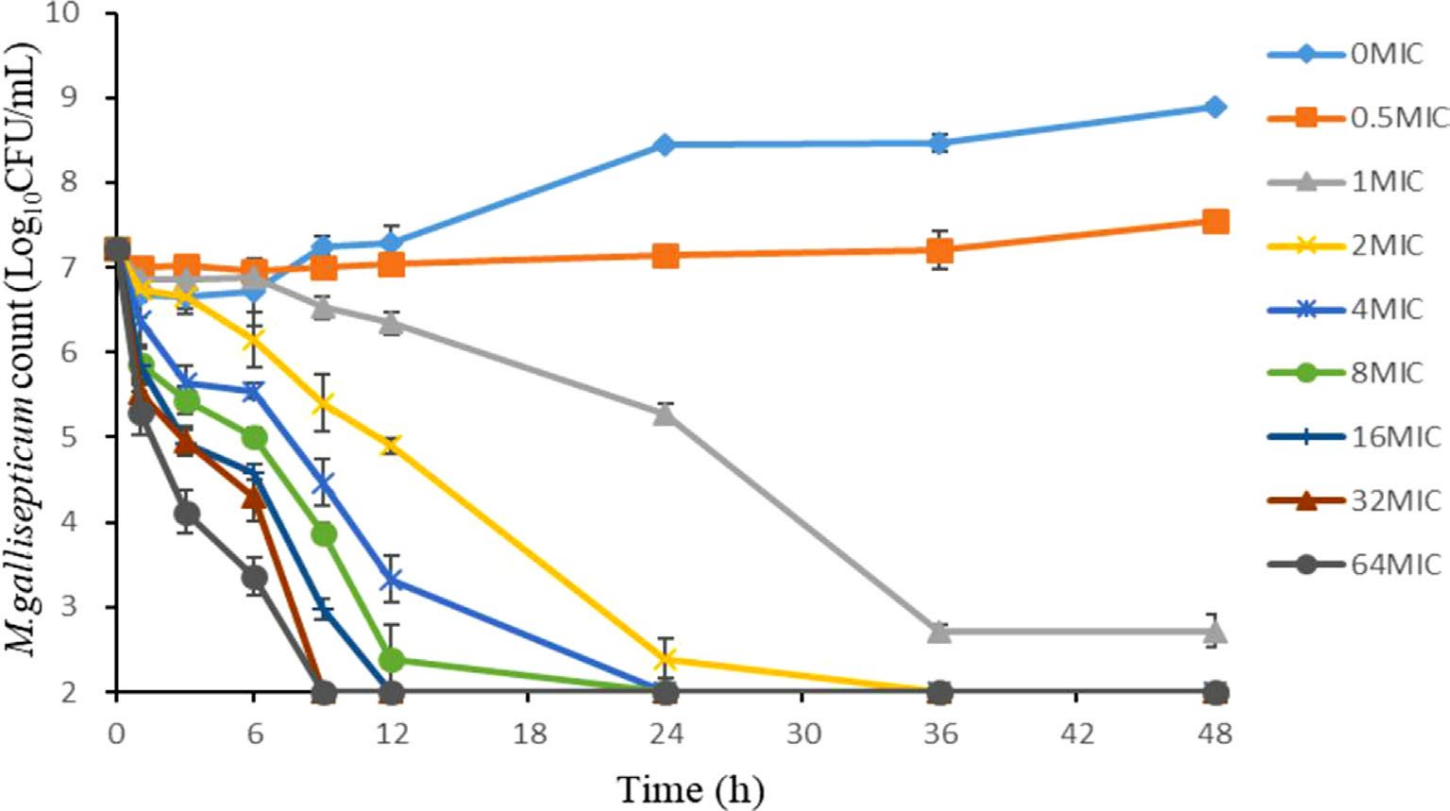
Time–killing studies of tilmicosin (as multiples of the MIC) against *M. gallisepticum* at constant concentrations. Data are presented as geometric means based on triplicates. MIC, minimum inhibitory concentration; CFU, colony forming units.

As shown in **Figure 2**, the static time-kill curve was in the form of a scatter plot. When the concentration exceeded 1 × MIC, tilmicosin had obvious antibacterial activity against *M. gallisepticum*. By contrast, when the concentration was lower than 1 × MIC, tilmicosin did not show significant antibacterial activity. At low concentration of tilmicosin (0.5 × MIC), *M. gallisepticum* counts only increased by 0.7 log_10_ (CFU/ml). However, when *M. gallisepticum* was exposed to 1–64 MICs for 48 h, the maximum reductions in bacterial counts were 4.24–4.89 log_10_ (CFU/ml), all of which achieved a bactericidal effect. Except for 0.5 × MIC and 1 × MIC concentrations, *M. gallisepticum* colonies decreased to the lowest detection limit (100 CFU/ml) after 48 h of tilmicosin treatment. On the whole, the antimycoplasma effect was more obvious as the drug concentration increased.

**Figure 3:**
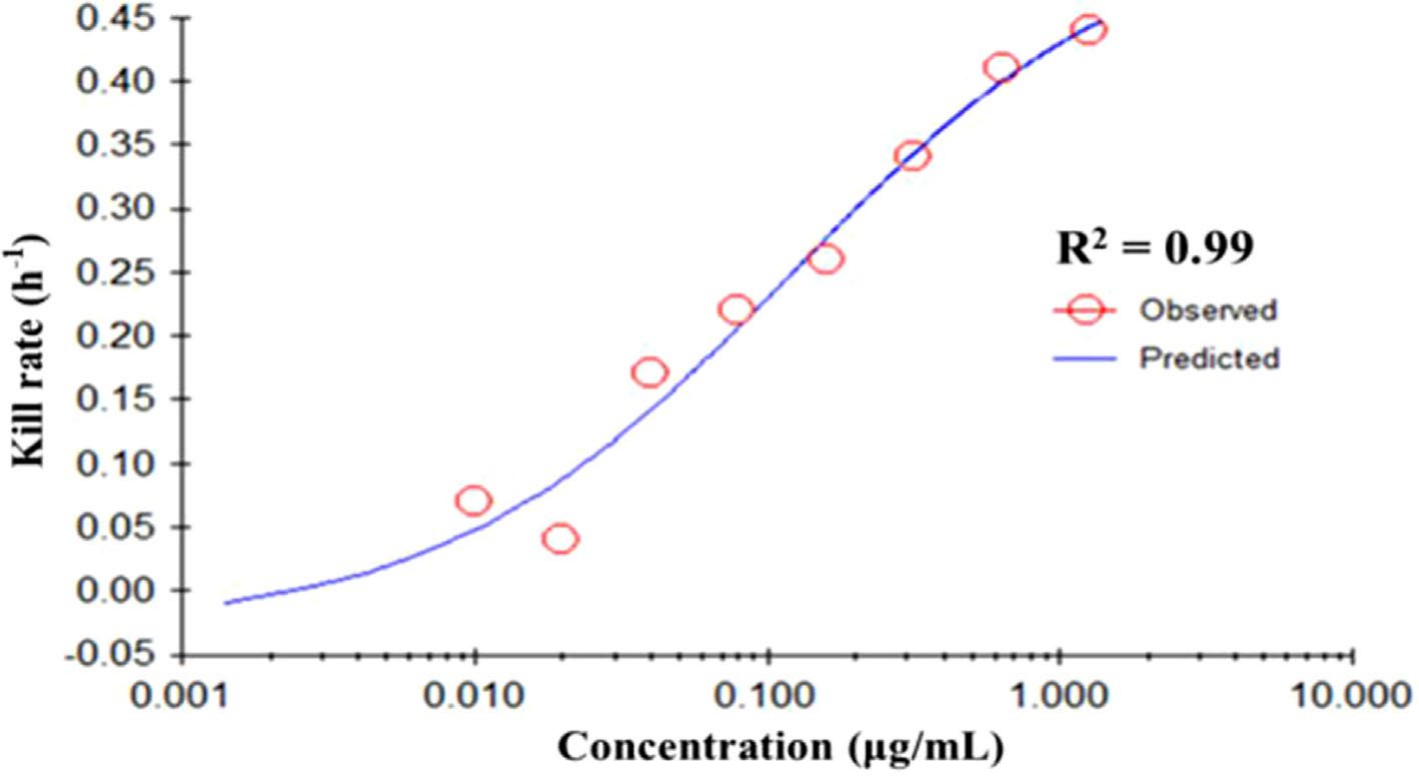
The best-fit curve obtained from the *E*_max_ model of *M. gallisepticum* exposed to tilmicosin between 0 and 48 h. *R* ^2^ is the correlation coefficient. *E*_max_, sigmoid maximum effect.

The fitting curve of drug concentrations and the kill rate is displayed in **Figure 3**. To reduce the error, the average kill rates of five time intervals were selected to represent the slope of the time-kill curve. With increasing drug concentration, the kill rate increased; however, the rate of increase slowed down slightly at higher concentrations. The correlation between drug concentration and the average kill rate was 0.99. The maximum kill rate was 0.53 h^-1^. Parameters (*E*_0_, *E*_max_, *EC* *_50_*, Hill coefficient) fitted by the *E*_max_ model are presented in **Table 1**.

**Table 1:** The kill rate parameter estimation derived from the *E*_max_ model which fitted to the *in vitro* static time–killing assay data.

*E*_max_ model parameter	Value
*E*_max_	0.53
EC_50_	0.12
*E*_0_	−0.03
Hill’s slope	0.73
*R* ^2^	0.99

**Figure 4:**
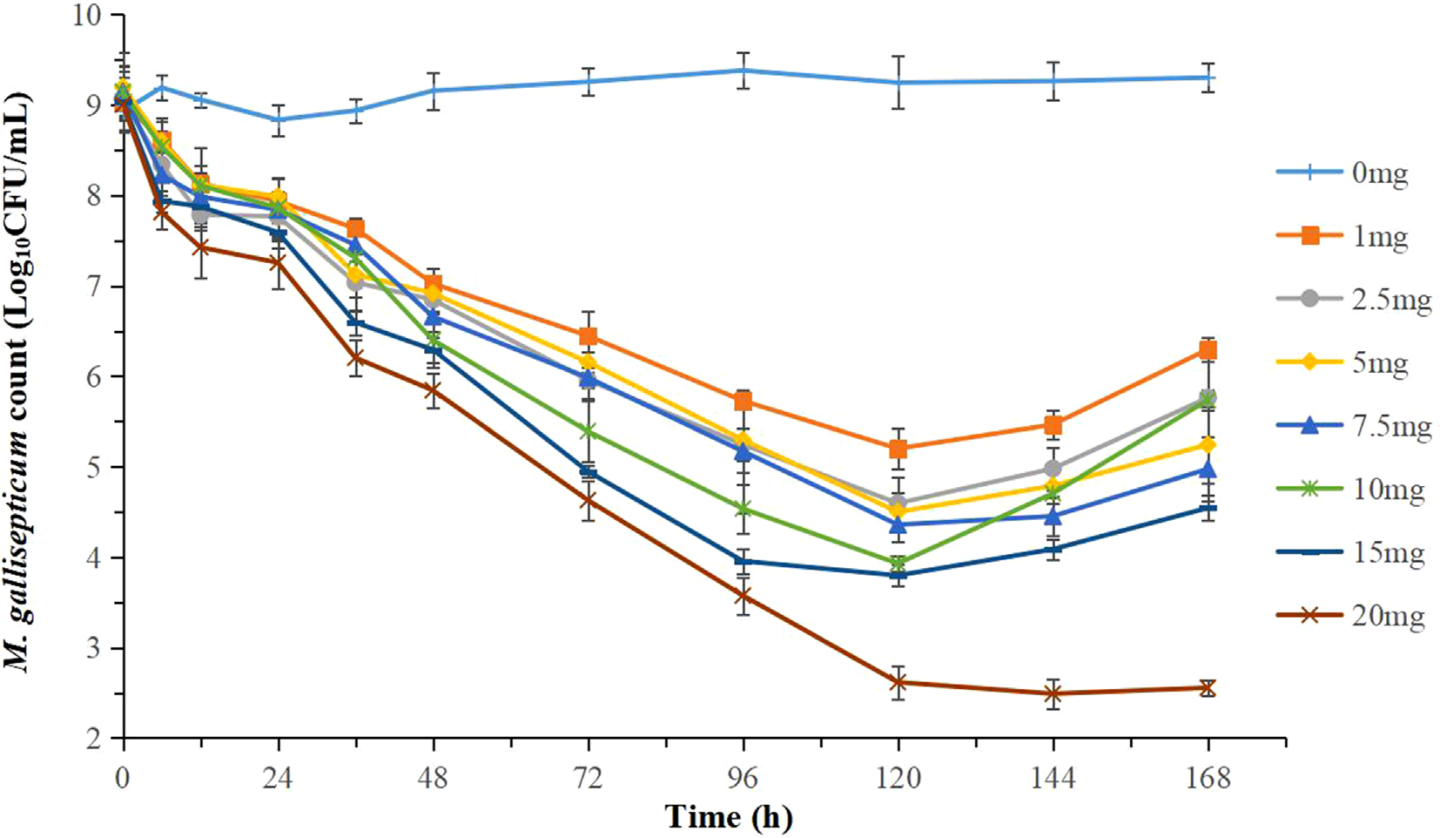
Dynamic time-killing curves were depicted at different concentrations of tilmicosin for the seven simulated doses. Data points represent geometric means of three experiments.

The effects of tilmicosin against *M. gallisepticum* at different clinically recommended doses in the dynamic *in vitro* model are presented in **Figure 4**. With escalating doses of drug, tilmicosin demonstrated increased activity against *M. gallisepticum*. Obvious bacteria regrowth was observed in all regimens except for the highest dose group after 120 h. The maximum *M. gallisepticum* count decreased from 3.30 to 5.43 Log_10_ CFU/ml when incubated in the constantly diluted drug for 94 h, achieving mycoplasmacidal activity.

### *In Vitro* Simulated Pharmacokinetics

**Figure 5:**
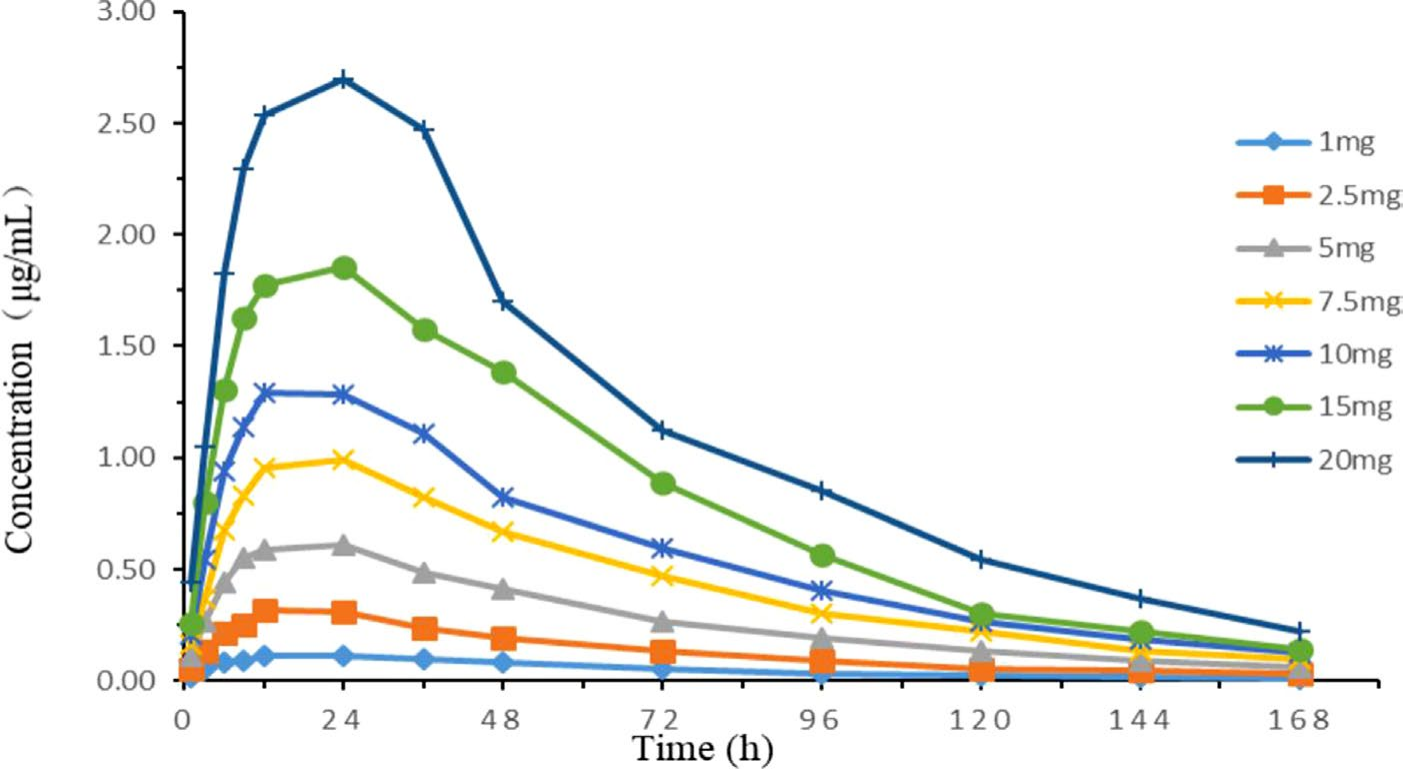
Concentration–time curves of seven doses of tilmicosin according to the lung pharmacokinetic data of chickens in the *in vitro* dynamic model.

Time-concentration curves simulating different clinical doses are shown in **Figure 5**. The PK parameters are summarized in **Table 2**. The relative errors of absorption half-life and elimination half-life were −6.18 and −3.08%, respectively, which were within the normal ±15% (and acceptable) range. The one-compartment open model with first-order absorption had a high correlation (*R* *^2^* > 0.99) with each dose group data.

**Table 2:** Main pharmacokinetic parameters of different tilmicosin dosages in the *in vitro* PK/PD model.

Pharmacokinetic parameters	Dose group (mg)							$\bar{X}$	Relative deviation %
	1	2.5	5	7.5	10	15	20		
D (mg)	0.089	0.223	0.446	0.670	0.893	1.339	1.786	–	–
t_1/2Ka_ (h)	5.24	4.71	4.66	5.63	5.16	5.80	5.58	5.25	6.18%
t_1/2Kel_ (h)	40.18	44.25	43.11	41.37	41.99	39.75	39.92	41.51	3.08%
T_max_ (h)	17.72	17.03	16.77	18.74	17.79	18.86	18.41	18.41	0.32%
C_max_ (µg/ml)	0.11	0.29	0.60	0.98	1.29	1.84	2.67	–	–
V_Central_ (mL)	574.95	584.19	563.50	500.08	515.99	487.29	485.34	530.19	6.04%
R^2^	0.997	0.993	0.999	0.997	0.998	0.996	0.997	–	–

Relative deviation = (PK parameters in vitro/PK parameters in vivo—1) * 100%; –, no data.

### Susceptibility Testing of *M. gallisepticum*

Seven *M. gallisepticum* strains (M1–M7) with different degrees of decreased susceptibility to tilmicosin were isolated from the seven dose groups. The changes in susceptibility for six antibacterial agents are presented in **Table 3**. The MIC of strains M1–M7 to tilmicosin ranged from 0.08 to 5.12 μg/ml. In particular, the MIC values of strains M3, M4, and M5 were significantly higher than that of strain S6. The seven strains of *M. gallisepticum* also acquired resistance to erythromycin and tylosin. However, no change of susceptibility for amikacin and doxycycline in these strains was observed. For tiamulin, lincomycin, and enrofloxacin, some strains showed decreased susceptibility.

**Table 3:** The MIC of six antimicrobial agents against *M. gallisepticum* S6 and M1–M7 strains.

Strain	MIC value of antibiotics (µg/ml)							
	Tilmicosin	Tylosin	Erythromysin	Tiamulin	Doxycyclin	Enrofloxacin	Amikacin	Lincomycin
S6	0.01	0.01	0.01	0.005	0.05	0.05	25.63	6.56
M1	0.16	0.16	0.08	0.005	0.05	0.05	25.63	6.56
M2	0.08	0.02	0.04	0.01	0.05	0.1	25.63	13.16
M3	5.12	0.16	>64	0.02	0.05	0.05	25.63	52.50
M4	0.64	0.08	>64	0.02	0.05	0.05	25.63	52.50
M5	1.28	0.16	>64	0.02	0.05	0.05	25.63	52.50
M6	0.16	0.04	0.16	0.02	0.05	0.1	25.63	13.13
M7	0.16	0.02	0.64	0.005	0.05	0.05	25.63	13.13

M1 (1 mg), M2 (2.5 mg), M3 (5 mg), M4 (7.5 mg), M5 (10 mg), M6 (15 mg), and M7 (20 mg) strains were selected from seven dosages, respectively.

### Pharmacokinetic/Pharmacodynamic Modeling and Analysis

**Figure 6:**
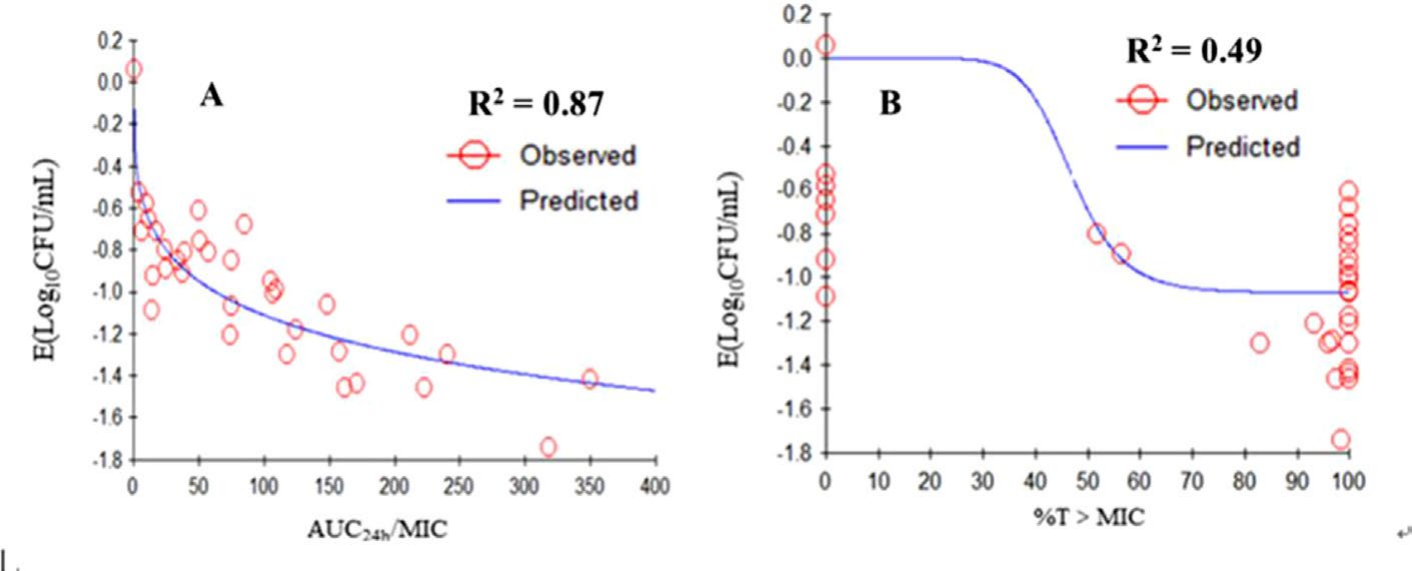
*E*_max_ relationships for the three PK/PD parameters versus the antimycoplasmal effect. **A.** AUC_0-24h_/MIC-antimycoplasmal effect curve; **B.** %T > MIC-antimycoplasmal effect curve. *R* ^2^ is the correlation coefficient. *E*_max_, sigmoid maximum effect; PK, pharmacokinetic; PD, pharmacodynamic; AUC, area under the concentration-time curve; MIC, minimum inhibitory concentration; %T > MIC is the cumulative time that the concentration exceeds the MIC.

The relationships between the PK/PD parameters deduced from dynamic model assays and antibacterial effect are shown in **Figure 6**. The correlation coefficients between AUC_24h_/MIC, %T > MIC, and the antibacterial effect were 0.87 and 49%, as analyzing using the inhibitory sigmoid *E*_max_ model. The results showed that AUC_24h_/MIC was the best-fit PK/PD parameter to predict the antimicrobial activity of tilmicosin against *M. gallisepticum*, which suggested that tilmicosin displayed concentration-dependent activity. The magnitude of AUC_24h_/MIC predicted for 1 Log_10_ (CFU/ml) reduction was 62.58 h during the 24-h treatment period of tilmicosin. The obtained parameters of *E*_0_, *E*_max_, and *EC* *_50_*, and the Hill coefficient are listed in **Table 4**.

**Table 4:** The PK/PD parameter estimation, and the data are derived from *E*max model.

PK-PD parameter	*E*_max_ (Log_10_CFU/mL)	EC_50_	*E*_0_ (Log_10_CFU/mL)	Hill’s slope	*R* ^2^
AUC_24h_/MIC(h)	2.98×10^-04^	849.68	−3.35	0.33	0.87
T>MIC (%)	4.00×10^-06^	46.93	−1.07	9.66	0.49

### Mutation Analysis

Gene mutation analysis was performed on the basis of annotated single nucleotide polymorphisms (SNPs), using the genome of strain S6 as the reference. As shown in **Table 5**, for strains M3, M4, and M5, no resistance mutations were found in the *rplD* and *rplV*, genes encoding ribosomal proteins L4 and L22. No mutations were detected in domains II and V of the 23S *rrnA* gene in strain M4. For strain M5, a G495T mutation occurred in domain II of the 23S *rrnA* gene; however, domain V of the 23S *rrnA* gene remained unchanged. For strain M3, a T854A mutation in domain II of the 23S *rrnB* gene and a G2799A mutation in domain V of the 23S *rrnB* gene were identified as associated with tilmicosin resistance.

**Table 5:** Mutations in the 23S rRNA gene and the ribosomal protein gene in M3, M4, and M5 strains.

Strains	MIC (µg/ml)	23S *rrnA*		23S *rrnB*		Ribosomal protein	
		II region	V region	II region	V region	rplD (L4)	rplV (L22)
S6 M3	0.01 5.12	– –	– –	– T854A	– G2799A	– WT	– WT
M4	0.64	WT	WT	–	–	WT	WT
M5	1.28	G495T	WT	–	–	WT	WT

–, No mutant was found.

WT, wild-type.

M3, M4, M5: mutants were selected from the dosages of 5 mg, 7.5 mg, and 10 mg, respectively.

## Discussion

*Mycoplasma gallisepticum*, which reduces egg production, hatchability, feed efficiency, and weight gain in chickens, has attracted particular attention in poultry disease research (Evans et al., 2005). Currently, vaccines or antibiotics are applied to prevent and control *M. gallisepticum* infections. A series of clinical symptoms or pathological changes are used to evaluate antimicrobial activity (Charleston et al., 1998). However, there is no information available on the PK/PD interactions of tilmicosin against *M. gallisepticum*.

Li et al. (2016) investigated the PK/PD relationship of cefquinome against *Staphylococcus aureus* using an *in vitro* PK/PD model, whereas this model was suited for the bacteria growing rapidly. To decrease the error of efficacy assessment caused by pumping out the medium, an *in vitro* PK/PD model can be set to change incrementally towards a dialysis model (Nan et al., 2016). Previous studies tested the dialysis model and showed that it overcame the above-mentioned error and also maintained a constant volume. Hollow-fiber (Tawanda et al., 2007; Gumbo and Dona, 2009) or semipermeable cellulose membranes (Al-Saigh et al., 2012; Meletiadis et al., 2012) were adopted for the *in vitro* dynamic model. In this study, a semipermeable cellulose membrane was used in consideration of the sterilization of the device and the simplicity of operation. We first reported an *in vitro* PK/PD model for *M. gallisepticum* that simulated the pharmacokinetics of tilmicosin in lung tissue, where *M. gallisepticum* colonizes after the single oral administration. The advantage of this model is that when it is difficult to establish an animal infection model, the influence of PK parameters obtained at different dosages on the drug’s effect can be clarified, and the change of drug sensitivity of pathogenic bacteria can be monitored, to enable further study of the drug resistance mechanism of bacteria.

The MICs of tilmicosin against strain S6 were similar to those reported by Zhang et al. (2017b), whether determined by the agar dilution method or the microdilution method. Compared with danofloxacin and doxycycline (Zhang et al., 2017b), the MIC value of tilmicosin is relatively low and the kill rate of danofloxacin (0.12 h^-1^) is far lower than that of tilmicosin (0.53 h^-1^) (Nan et al., 2016), which shows that *M. gallisepticum* is more sensitive to tilmicosin. There are two main reasons for choosing the high inoculum (10^9^ CFU/ml). First, tilmicosin accumulates mainly in the lung, leading to a much higher concentration in lung tissue than in plasma. Second, the mutant subpopulations are present at low frequencies (10^-6^ to 10^-8^) (Drlica and Zhao, 2007). Therefore, the high inoculum could increase the probability of monitoring mutant strains. The design of the inoculum met the requirement for assessing efficacy and the risk of drug resistance.

For the macrolides, most studies have shown that the %T > MIC parameter was significantly correlated with antimicrobial activity (Carbon, 1998; Fran et al., 2004). Nevertheless, the antibacterial activity of azithromycin, with a long elimination half-life, was related to the AUC_24h_/MIC parameter (Van and Tulkens, 2001). Antibacterial activity is determined by the characteristics of the drug and the bacteria. When the concentration of a bacteriostatic drug is high enough, it can also show bactericidal activity (Piscitelli et al., 1992). Therefore, drugs may exhibit different characteristics against diverse bacteria. The results of the present study showed that the AUC_24h_/MIC parameter had the highest correlation with the antibacterial effects (*R* *^2^* = 0.87). Therefore, we could infer that the antimicrobial effect of tilmicosin against *M. gallisepticum* was concentration-dependent. Tilmicosin is slowly eliminated in animals and generates drug persistence in lung tissue; therefore, the effective antibacterial concentration could be maintained for several days after a single dose. In addition, the MIC value *in vitro* was relatively small. These observations might explain why the %T > MIC was not the optimal parameter.

The mutant selection window (MSW) hypothesis states that when the drug concentration falls between the MIC and the mutant prevention concentration (MPC), drug–resistance mutants will be selectively enriched (Drlica and Zhao, 2007). Zhang et al. (2017b) reported that the concentration range of the MSW for tilmicosin against *M. gallisepticum* was 0.027–0.15 μg/ml. All dose groups used in the present study fell within the MSW, which may have resulted in reduced sensitivity strains being screened. The test results showed that strains with reduced sensitivity were screened at all recommended doses, and these strains had cross-resistance to the same class of drugs. Thus, when a strain is resistant to tilmicosin, using similar drugs may lead to treatment failure, and other classes of drugs should be considered for treatment.

**Figure 4** showed that the regrowth of *M. gallisepticum* was observed after 120 h in the dynamic model. The reasons were mainly as follows. First, the drug concentration was gradually diluted to near the MIC. Second, the susceptibility of *M. gallisepticum* changed under the sustained action of tilmicosin. Currently, reports on the resistance mechanism of mycoplasma to macrolides are limited to the mutation of drug target molecules and the efflux of antimicrobial active substances. For strains (M4 and M5) with remarkable changes in sensibility, further study found that the T854A and G495T mutations in domain II, and the G2799A mutation in domain V of the 23S rRNA gene were associated with resistance. Tilmicosin-resistant *M. gallisepticum* strains isolated from animals or induced *in vitro* had mutations at positions 2057, 2058, or 2059 in the V region of the 23S rRNA gene (Gerchman, 2011; Ammar et al., 2016). However, the strain obtained in our experiment was not mutated at these reported sites, which may be related to the route or mode of obtaining the strain. Most of the previously analyzed resistant strains were isolated from animals. The resistance of the strains was likely affected by the complex environment in which they were located and the actual conditions of the animals.

One of our study limitations was that the plasma protein binding rate of tilmicosin was not taken into account in *in vitro* experiments. The plasma protein binding rate of tilmicosin on sheep was 16.8% (Zhang et al., 2016). According to the PK studies of tilmicosin (Morck et al., 1993; Zhang et al., 2017a), the serum drug concentration was lower than the MIC required for the *M. gallisepticum* of 10^9^ CFU/ml, which was not a reasonable reference for the experiment, and the concentration of tilmicosin in plasma was much lower than that in lung tissue. We simulated drug concentrations in the lungs, and did not consider plasma protein binding rate. The second limitation was that all experiments were performed under ideal conditions *in vitro*, and the effect of animal’s immune system on the microorganism could not be taken into account.

In conclusion, the results of the present study indicated that when drug concentrations were constant, tilmicosin produced a maximum antimycoplasma effect of a 4.89 log_10_ (CFU/ml) reduction. Moreover, the kill rate analysis not only accurately quantified the antibacterial effect of tilmicosin against *M. gallisepticum* compared with using the MIC alone, but also verified that the antibacterial effect of tilmicosin was concentration-dependent. At the same time, the *in vitro* dynamic model test also showed that the antimycoplasma activity of tilmicosin against *M. gallisepticum* was concentration-dependent and the best-fit PK/PD parameter was AUC_24h_/MIC (R^2^ = 0.87). The magnitude of AUC_24h_/MIC predicted for 1 Log_10_ (CFU/ml) reduction was 62.58 h, and the bactericidal effect can be achieved within 3 days when the simulated dose group ≥ 7.5 mg dose group (administered dose ≥ 0.067 mg). These data provided a reliable basis for *in vivo* experiments and may help to design a more rational treatment regimen for *M. gallisepticum* infection.

## Data Availability Statement

The raw data supporting the conclusions of this manuscript will be made available by the authors, without undue reservation, to any qualified researcher.

## Author Contributions

Methodology, software, validation, formal analysis, data curation, manuscript preparation, manuscript reviewing and editing, visualization, and project administration were performed by ZH. ZH, YW, ZZ, and XX contributed to the investigation. Resources were provided by XG, QC, XS, HY, and HD. Supervision was provided by HD, who also acquired the funding.

## Conflict of Interest Statement

The authors declare that the research was conducted in the absence of any commercial or financial relationships that could be construed as a potential conflict of interest.
